# The Strengths and Difficulties Questionnaire Parent Form: Dutch norms and validity

**DOI:** 10.1186/s12887-022-03274-6

**Published:** 2022-04-12

**Authors:** Meinou H. C. Theunissen, Marianne S. de Wolff, Iris Eekhout, Cathelijne L. Mieloo, Lisanne L. Stone, Sijmen A. Reijneveld

**Affiliations:** 1 TNO Child Health, P. O. Box 3005, Leiden, 2301 DA the Netherlands; 2 The Hague University of Applied Sciences, The Hague, The Netherlands; 3 Karakter, Child and Adolescent Psychiatry University Center Nijmegen, Nijmegen, The Netherlands; 4 Department of Health Sciences, University Medical Center Groningen, University of Groningen, Groningen, The Netherlands

**Keywords:** SDQ, Emotional and behavioral problems, Children, Community pediatric services, Norms

## Abstract

**Objective:**

This study provides Dutch national norms for the parent-reported Strengths and Difficulties Questionnaire (SDQ) for children aged 3-14 years, and assesses the test performance of the SDQ Total Difficulties Scale (TDS) and impairment Scale. We further compared Dutch SDQ norms with those of the United Kingdom (UK), to determine potential variation in country-specific norms.

**Study design:**

We analyzed data of 3384 children aged 3 to 14 years. The data were obtained in schools, and in the context of Preventive Child Healthcare. Parents completed the SDQ parent form and the Child Behavior Checklist (CBCL). We determined clinical (10% elevated scores) and borderline (20% elevated scores) SDQ TDS norms. We assessed the test performance (validity) of the SDQ TDS and Impairment Score using the CBCL as criterion.

**Results:**

The clinical SDQ TDS norms varied between > 10 and > 14 depending on the age group. The SDQ TDS discriminated between children with and without problems, as measured by the CBCL, for all age groups (AUCs varied from 0.92 to 0.96). The SDQ Impairment Score had added value (beyond the SDQ TDS) only for the age group 12-14 years. For the Netherlands we found lower clinical SDQ TDS norms than those previously reported for the UK (i.e. > 16).

**Conclusion:**

The clinical SDQ TDS norms varied between > 10 and > 14 depending on the age groups. We found good test performance at these proposed norms. Dutch norms differed somewhat from UK norms. In the Netherlands, the SDQ performed better with Dutch-specific norms than with UK-specific norms.

## Introduction

Many children have symptoms of mental health problems such as low self-esteem, depressive thoughts, impulsive or maladaptive behaviors [1, 2]. These Emotional and Behavioral Problems (EBP) can negatively impact a child’s development and later evolve into serious mental health problems [3, 4]. Early recognition and identification of EBP can improve the prognosis of these children [5–7].

Community pediatric services can play a major role in the identification of EBP in children, and this role can be enhanced by adequate tools. In the Netherlands, Preventive Child Healthcare (PCH) routinely offers preventive health care to all children aged 0-18 years, similar to well child care in the USA. PCH should preferably be supported by validated and normed questionnaires for the identification of EBP. Validated short questionnaires have been shown to support the quality of identification [8, 9]. The Strengths and Difficulties Questionnaire (SDQ), currently one of the most widely used behavioral screening questionnaires [10], is a major candidate for such a supporting role. It is available in many languages, and in two age versions: the SDQs 3-4 and 4-16. The SDQ can be completed by parents, teachers, and the youngsters themselves. The SDQ consists of items related to a child’s strengths (pro-social behavior scale) and difficulties (Total Difficulties Scale, TDS), and items related to the severity and the impact of problems; the latter is also called the Impairment Scale. The psychometric properties of the SDQ Parent Form (SDQ-PF) have been shown to be good in various settings and countries [9, 11–14], including the Netherlands [15–22].

The availability of Dutch validated norms for the SDQ-PF is limited in terms of age coverage and of national representativeness of the sample [23]. Only one study covered a wider age range using a national sample, but this study did not address the test-performance of the SDQ at the proposed norms [24]. In the UK, community-based SDQ norms exist for the Parent Form, covering a wide age range (4-17 years) [25]. However, it is unknown whether these UK norms are also suitable for other West-European countries, like the Netherlands. Moreover, evidence is needed on the added value of including the SDQ Impairment Scale along with the TDS. PCH professionals indicate that the Impairment Scale is highly valuable for the identification of EBP; however, until now, the added value of the Impairment scale has been assessed only for ages 3-4 years [26] and 7-12 years [18].

The aim of this study was therefore to provide Dutch national norms for the parent-reported SDQ regarding a wide age-range relevant for PCH (3-14 years), and to assess the test performance of the SDQ TDS and Impairment Scale at the normed cut-offs. We further compared the Dutch community-based SDQ norms with UK norms to determine possible country-specific variations.

## Methods

To assess the validity of the SDQ-PF we combined data from five studies conducted in the Netherlands during the period 2003-2011. We obtained data from schools (study 2) and in the context of routine PCH assessments that are provided regularly for all Dutch children (studies 1, and 3-5). Parents completed both the SDQ-PF and the questionnaire that we used as a criterion: the Child Behavior Checklist (CBCL). We obtained ethical approval for these studies from the Medical Research Ethics Committee (METC) of Leiden University Medical Center (studies 1, 4, and 5), the METC of the Radboud University Nijmegen (study 2) and the METC of the Erasmus University Medical Center Rotterdam, the Netherlands (study 3).

### Datasets

Data came from the following studies:

1. *SDQ 3-4 years.* A cross-sectional national study conducted in 2008-2011 (ages 36-45 months; 839 parents participated, response rate 65.5%) [26].
2. *SDQ 4-7 years.* A longitudinal national study (3 waves) conducted in school year 2009-2010 (grades 1 to 4, ages 4 to 7 years; 1513 parents out of approximately 2300 participated). For this study we used baseline data [21].
3. *SDQ 4-7 years*. A cross-sectional national study conducted in the school year 2008-2009 (grade 2, ages 5-6 years. 8114 parents participated, response rate 67%). In addition to the SDQ, 801 parents filled out the Child Behavior Checklist (CBCL) [23].
4. *SDQ 7-11 years.* A cross-sectional national study conducted in 2003-2004 (ages 7-11 years; 711 parents participated, response rate 87%) [18].
5. *SDQ 12-14 years*. A cross-sectional national study conducted in 2009-2010 (ages 12-14 years; 602 parents participated, response rate 62.6%) [27].

### Measurements

For each study we used the parent version of the SDQ (SDQ-PF) [10, 15, 16]. We used the SDQ-PF 3-4 (study 1, covering ages 3 and 4 years) and SDQ-PF 4-17 (studies 2-5, covering ages 4 to 17 years). Both questionnaires consist of 25 items related to a child’s strengths and difficulties and 8 items related to the severity and impact of problems. Each strengths and difficulties item is scored on a 3-point scale (0 = not true, 1 = somewhat true, and 2 = certainly true). The SDQ consists of five subscales, four on difficulties (emotional symptoms, conduct problems, hyperactivity-inattention, and peer problems) and one on strengths (pro-social behavior). An SDQ TDS can be calculated by adding up the scores on the difficulties subscales. The 8 questions on the impact of problems refer to duration, distress, social impairment, and burden for others. An Impairment Score was calculated by aggregating the scores for distress and social impairment. A 3-point scale was used for each impairment item: 0 = not at all/ only a little, 1 = quite a lot, and 2 = a great deal.

The CBCL was used as a golden standard for parent reports about the child’s emotional and behavioral problems over the preceding 6 months [28–31]. This included three slightly different versions: the CBCL/1.5-5 in studies 1 and 2, the CBCL/6-18 in studies 2, 3 and 5, and the CBCL/4-18 in study 4. The CBCLs contain either 120 problem items (CBCL/4-18; CBCL/6-18) or 100 problem items (CBCL/1.5-5), which are combined with a Total Problem Score (TPS) and with internalizing and externalizing problem scores, the latter two representing emotional and behavioral problems, respectively. Children were allocated to a normal range or an elevated range by using 90th percentile age and sex-specific cutoff points. For the older CBCL version (CBCL 4-18) in study 4 the Dutch norms were used [31].

Socio-demographic characteristics included the child’s age and gender, ethnicity, parental educational level, and family composition. In accordance with the classification system used by Statistics Netherlands, we classified a child as being Dutch when both parents had been born in the Netherlands. A child’s ethnic background was defined as non-Dutch when one or both parents had been born outside the Netherlands. Family composition indicates the number of parents in the family (2 parents or 1 parent).

### Procedure

Studies 1, 4 and 5 used the same two-step data collection procedure. First, PCH services were asked to participate. Second, PCH services that agreed to participate were asked to provide data about a specified number of children at specified ages. For study 2 a total of 440 schools schools were randomly selected from all elementary schools in the Netherlands. Of these, 29 (6.6%) cooperated in this study. For study 3 the SDQ data were collected in the context of the routine PCH assessment of grade 2 in the Rotterdam-Rijnmond area in the Netherlands. Parents of a sub-sample of children were also invited to fill out the CBCL in addition to the SDQ.

For studies 1, 3, 4, and 5 the data were collected within the context of routine PCH assessments. The SDQ and CBCL were mailed to parents along with the standard invitation for the well-child examination, and were filled in at home. The completed questionnaires were returned to the PCH professional in a sealed envelope and forwarded to the research institute without being opened. However, in study 3 the PCH professional used the SDQ during the assessment. The PCH professional provided data about child age and gender, family composition, and parental educational level. For study 2 the SDQ data were collected in a school setting; the questionnaries were filled out online by parents.

### Analyses

To answer our research questions we used data from a total of 3384 children. We excluded children with non-Dutch ethnicity because the share of these children was too small to allow for sub-analyses, and in non-Dutch children EBP problems are more prevalent [32, 33]. Consequently, our community-based norms apply only to children with Dutch ethnicity.

We first computed the background characteristics of each sample. Second, we determined the community-based norms. Children were allocated to a normal or an elevated range on the SDQ-PF TDS, using as cut-off the score that was associated with a percentage of elevated scores of 10% (elevated) or 20% (borderline), as has been done in other questionnaires that address psychosocial problems like the ASEBA-system [28, 30]. Third, we assessed the psychometric properties of the SDQ at the normed cut-offs. We assessed *internal consistency* using Cronbach’s alpha. We assessed *validity* as the agreement between the SDQ TDS and subscales scores and the CBCL scores (CBCL TPS, CBCL Internalizing and Externalizing problem scores), using Spearman correlation coefficients. We further assessed the validity of the SDQ TDS with sensitivity and specificity indices, using CBCL TPS as the criterion.

Finally, we determined the *added value* of the SDQ Impairment Score by assessing the degree to which the SDQ Impairment Score can improve identification of children with problems based solely on the SDQ TDS score. To do this we used four separate logistic regression analyses, with the CBCL TPS criterion as dependent variable. First we included the elevated SDQ TDS as independent variable, second the elevated SDQ Impairment Score, third a combination score of an elevated SDQ TDS **or** Impairment Score, and fourth a combination score of an elevated SDQ TDS **and** Impairment Score as independent variable. For each analysis we determined the Odds Ratios (OR) and sensitivity and specificity indices.

## Results

### Demographics

Table 1 presents demographic information about the study population.

**Table 1:** Background characteristics of the various samples, categorized by age

	3-4 years	4-7 years^**a**^	7-11 years	12-14 years
	*N* = 689	*N* = 1505	*N* = 656	*N* = 534
	n (%)	n (%)	n (%)	n (%)
Gender				
Boy	349 (50.7)	790 (52.5)	319 (48.8)	214 (39.9)
Girl	340 (49.3)	714 (47.5)	335 (51.2)	323 (60.1)
Child’s age				
2 years	79 (11.6)			
3 years	544 (79.8)			
4 years	59 (8.7)	304 (20.2)		
5 years		547 (36.3)		
6 years		403 (26.8)		
7 years		251 (16.7)	19 (2.9)	
8 years			78 (11.9)	
9 years			96 (14.6)	
10 years			181 (27.6)	
11 years			155 (23.6)	
12 years			108 (16.5)	10 (2.4)
13 years			19 (2.9)	201 (49.1)
14 years				198 (48.4)
Family composition				
Two parents	655 (96.5)	1107 (89.1)	559 (88.9)	466 (93.6)
One parent	24 (3.5)	106 (8.5)	70 (11.1)	32 (6.4)
Paternal educational level				
Lower education	145 (21.5)	NA	13 (2.2)	91 (21.6)
Medium education	279 (41.3)	NA	361 (60.9)	150 (35.5)
Higher education	251 (37.2)	NA	219 (36.9)	181 (42.9)
Maternal educational level				
Lower education	125 (18.3)	242 (17.8)	13 (2.1)	107 (25.0)
Medium education	321 (47.1)	566 (41.7)	436 (69.0)	171 (40.0)
Higher education	236 (34.6)	550 (40.5)	183 (29.0)	150 (35.0)

*NA* Not available

^a^Data on age group 4-7 come from two studies

### Community-based norms

Table 2 present the SDQ TDS clinical and borderline norms per sample. The SDQ TDS cut-off values varied by age group between > 10 and > 14 for the clinical SDQ TDS scores (10% elevated scores), and between > 7 and > 10 for the borderline SDQ TDS scores (20% elevated scores).

**Table 2:** Internal consistency (Cronbach’s alphas) of scores on the SDQ Total Difficulties scale and subscales, and test characteristics of the SDQ Total Difficulties using the CBCL Total Problems score as criterion

	3-4 years	4-7 years	7-11 years	12-14 years
**SDQ norms**				
Clinical SDQ score				
Cut-off point	> 10	> 14	> 13	> 11
% children with elevated SDQ TDS score	10.0	9.2	9.0	10.6
Borderline SDQ score				
Cut-off point	> 7	> 10	> 10	> 8
% children with elevated SDQ score	20.6	21.1	17.7	21.3
**Cronbach’s alpha**				
SDQ scales				
Total difficulties	0.78	0.79	0.80	0.77
Emotional symptoms	0.52	0.74	0.71	0.69
Conduct problems	0.63	0.54	0.56	0.39
Hyperactivity	0.75	0.77	0.78	0.76
Peer problems	0.52	0.49	0.55	0.63
Prosocial	0.65	0.83	0.67	0.75
**Validity indices**				
Clinical SDQ score				
Sensitivity	0.70	0.71	0.75	0.58
Specificity	0.94	0.95	0.96	0.95
Borderline SDQ score				
Sensitivity	0.84	0.90	0.92	0.81
Specificity	0.80	0.83	0.88	0.86
AUC (95% confidence interval)	0.92 (0.88-0.95)	0.94 (0.93-0.96)	0.96 (0.95-0.98)	0.93 (0.91-0.95)

*TDS* Total difficult scale, *AUC* Area under the curve

### Scale structure

Table 2 shows that Cronbach’s alphas varied between 0.77 and 0.80 for the SDQ TDS, between 0.75-0.78 for the SDQ subscale Hyperactivity, and between 0.39-0.83 for the other SDQ subscales, with variation occurring both by age and by scale. The internal consistencies, indicated by Cronbach’s alphas, of some of the SDQ subscales were relatively low and internal consistency is considered to be a prerequisite for validity [34]. Therefore, the assessment of the sensitivity and specificity indices were restricted to the SDQ TDS.

### Validity

The SDQ TDS and the SDQ subscale scores correlated significantly with the CBCL scores (Table 3). The correlation coefficients between the SDQ TDS and CBCL TPS varied from 0.69 to 0.81, between the SDQ Emotional symptoms score and the CBCL Internalising problems score from 0.43 to 0.71, and between the SDQ Conduct problems score and the CBCL Externalising problems from 0.54 to 0.69.

**Table 3:** Spearman correlations between scores on SDQ (sub)scales and CBCL Total, Internalizing, and Externalizing problems scores

	3-4 years			4-7 years			7-11 years			12-14 years		
	CBCL			CBCL			CBCL			CBCL		
SDQ scales	Total	Intern	Extern	Total	Intern	Extern	Total	Intern	Extern	Total	Intern	Extern
Total difficulties	0.69	0.56	0.66	0.81	0.60	0.68	0.69	0.53	0.57	0.73	0.53	0.58
Emotional symptoms	0.37	0.43	0.23	0.60	0.71	0.35	0.59	0.69	0.39	0.54	0.59	0.37
Conduct problems	0.59	0.41	0.64	0.57	0.33	0.65	0.49	0.28	0.59	0.47	0.24	0.59
Hyperactivity	0.52	0.34	0.58	0.57	0.29	0.50	0.04	0.19	0.39	0.05	0.20	0.42
Peer problems	0.30	0.33	0.21	0.46	0.41	0.34	0.04	0.37	0.24	0.03	0.40	0.20
Prosocial	−0.36	− 0.27	− 0.37	− 0.31	−0.21	− 0.35	−0.24	− 0.16	−0.24	− 0.36	−0.28	− 0.40

All correlations are *P* < 0.01

Table 2 presents the Area Under the Curve (AUC) and the sensitivity and specificity indices, using the CBCL TPS as criterion. Sensitivity and specificity for the SDQ TDS (clinical score) varied from 0.58 to 0.75, and from 0.94 to 0.96, respectively, using the CBCL TPS as criterion (Table 2, lower section). Repetition of these analyses with SDQ TDS borderline cut-off points yielded higher sensitivities (varying from 0.81 to 0.92) and lower specificities (varying from 0.80 to 0.88).

### Added value

Table 4 shows that for age groups 3-4, 4-7 and 7-11 years a single elevated SDQ TDS score results in a better sensitivity (ranging from 0.70 to 0.75) and specificity (ranging from 0.95 to 0.96) than either a single elevated SDQ Impairment Score or the combination of both TDS and Impairment scores. However, for the age group 12-14 years, the sensitivity of the SDQ TDS alone was rather low (0.58) at a high specificity (0.95). The sensitivity and specificity indices for this age group were optimal when using the combination of an SDQ TDS and an SDQ Impairment Score (one **or** both elevated) (sensitivity = 0.85, and specificity = 0.89).

**Table 4:** Results of separate logistic regression analyses: on elevated CBCL TPS as outcome measure, and SDQ TDS and SDQ Impairment Score as predictors

	SDQ 3-4			SDQ 4-7			SDQ 7-11			SDQ 12-14		
	CBCL			CBCL			CBCL			CBCL		
	OR (95%CI)	Sens.	Spec.	OR (95%CI)	Sens.	Spec.	OR (95%CI)	Sens.	Spec.	OR (95%CI)	Sens.	Spec.
Elevated SDQ TDS score	60.0 (30.7-117.3)	0.70	0.94	43.5 (28.2-67.2)	0.71	0.95	83.0 (38.7-178.2)	0.75	0.96	23.9 (12.0-47.5)	0.58	0.95
Elevated SD Impairment score	41.0 (19.0-88.6)	0.42	0.98	24.3 (14.5-40.9)	0.89	0.76	21.9 (11.4-42.2)	0.71	0.90	19.4 (10.1-37.6)	0.62	0.92
Elevated SDQ TDS **or** Impairment score	57.0 (29.4-110.5)	0.74	0.95	53.5 (24.8-115.5)	0.95	0.73	61.8 (28.4-134.5)	0.89	0.88	45.5 (20.2-102.3)	0.85	0.89
Elevated SDQ TDS **and** SDQ Impairment score	94.4 (31.4-284.0)	0.38	0.99	55.8 (35.4-87.9)	0.68	0.96	61.8 (28.4-134.5)	0.58	0.98	23.0 (9.9-53.8)	0.35	0.98

## Discussion

This study aimed to provide Dutch national norms for the parent-reported SDQ regarding a wide age range (3-14 years) in routine community-based settings, to assess its psychometric properties and the added value of the SDQ Impairment Score. Our findings show that clinical SDQ TDS norms depended on age group, and varied between > 10 and > 14 (10% elevated scores); for the borderline SDQ TDS, norms (20% elevated scores) varied between > 7 and > 10. The SDQ TDS discriminates between children of all age groups, with and without problems, as measured by the CBCL TPS. The SDQ Impairment Score had added value (compared to the SDQ TDS) only for the age group 12-14 years, and not for ages 3-11 years. We further compared the Dutch community-based SDQ norms with the United Kingdom (UK) norms, to determine whether country-specific norms differ.

### Interpretation

We found lower clinical SDQ TDS norms (i.e. > 10 to > 14) than previously reported, especially for children aged 7 years and over, which were previously reported as varying from > 14 to > 17 [24]. This may be due to several factors. First, our data were collected in the context of routine PCH assessments, i.e. in a fully community-based care setting that includes the entire population. By contrast, the data of Stam et al. [24] came from a panel setting of a for-profit agency, i.e. a selection of people who agreed to participate in a series of surveys that have no further consequences. The latter type of respondents and setting may lead to more open disclosure and thus higher problem scores. Second, we excluded children with a migrant background, whereas Stam et al. [24] did not. This may have lowered the SDQ scores, and consequently the SDQ norms in the current study, because EBP problems are more prevalent in non-Dutch children [32, 33]. Our results may therefore be more valid in a preventive setting for native Dutch children.

We found lower clinical SDQ TDS norms (i.e. > 10 and > 14) for the Netherlands than previously reported for the UK (i.e. > 16) [25], with greatest differences for age groups 3-4 and 12-14 years. This may reflect either sampling differences or actual differences between the Netherlands and the UK. Regarding sampling differences, the current study involved a representative community-based national sample of Dutch-born children, whereas the representativeness of the UK sample is unclear [25]. Regarding actual differences between the Netherlands and the UK, the lower Dutch SDQ norms may reflect a lower prevalence of SDQ problems. National data on the well-being of children support this difference, indicating that youth well-being is higher in the Netherlands than in the UK [35]. At least some real differences seem likely, thus warranting the use of setting-adapted norms.

We generally found the Dutch version of the SDQ-PF to have good psychometric properties across a wide age-range, confirming findings of previous research in the Netherlands [19, 21, 23, 26, 27] that also align with findings on other countries [36, 37]. However, the internal consistencies of some subscales were relatively low, again confirming findings on other countries [37]. This may be partly due to the small number of items per scale (5 items). These low internal consistencies of the SDQ subscales indicate that they are inappropriate to be used to decide whether individual children require further attention. However, the sensitivities and the AUC (which measure the accuracy of the SDQ for detection of problems) of the SDQ TDS, are in the same range with previous studies and show good validity indices. The validity indices are better at the proposed norms compared to UK (> 16) norms. The good test performance of the SDQ TDS at the Dutch normed cut-offs justifies the use of these proposed norms.

We found the SDQ PF TDS to have a high AUC (0.93) for ages 12-14 years, indicating that the SDQ discriminates as well for these ages as for other ages. However, at the proposed norm for this particular age group we found a relatively poor sensitivity (0.58), but a high specificity of 0.95. An explanation for this high AUC but relatively low sensitivity may be that the proposed cut-off (10% elevated scores) is not optimal, due to chance. This seems indeed to be the case; at a cut-off of 20% elevated scores the sensitivity is 0.81, at a specificity of 0.86. Although this suggests that the SDQ-PF is a valid instrument for ages 12-14 years, for this particular age group a lower norm should be considered.

Finally, we found that the SDQ Impairment Score had no added value (compared to the SDQ TDS), except for ages 12-14 years. This finding is in line with previous studies that showed that the SDQ Impairment Score had no added value among 3-4 year olds [26] and 7-11 year olds [18]. In these studies, the contribution of the SDQ Impairment Score overlaps with the SDQ TDS score. The finding that the SDQ Impairment Score has added value for ages 12-14 years may be explained by the relatively poor sensitivity of the TDS score, as discussed above. In light of this inadequacy of the SDQ TDS score for ages 12-14, the SDQ Impairment Score may add information for the identification of EBP.

### Strengths and limitations

Our study has a number of strengths, such as its large sample size, community-based nature, and moderate to high response rate, thereby limiting the likelihood of selective response and increasing its ecological validity. Furthermore, 4 out of 5 studies covered the entire Netherlands. Another strength is that our analysis included an ethnically homogenous population. Consequently, we present community-based norms only for children with a Dutch ethnicity. A limitation may be that we used data collected in 2003-2010 to determine the SDQ norms. However, this is unlikely to have a large effect on the SDQ norms, because the SDQ has not been modified in any way since its introduction, either in English or in other languages.

### Implications

Our findings imply that the Dutch country-specific norms have added value, as they differ from UK norms, and that using these Dutch norms the psychometric performance of the SDQ is good, similar to previous findings on the SDQ Self report [27]. These Dutch norms should thus be preferred for use in the Dutch PCH setting. Preferably, they should be further validated by comparing them with psychiatric diagnoses as set, similar to approaches on previous cut-offs [38].

We found different SDQ norms for the various age groups. This finding implies that age-specific SDQ norms should be considered. The same may also apply to UK norms, which are now the same for age groups 5-10 and 11-15 years. However, regarding this issue further study is evidently required.

Our conclusion is that age- and country-specific SDQ norms indeed have added value. This finding requires confirmation in other countries and/or other ethnic groups. Moreover, optimal cut-offs can greatly enhance both research and care based on this well-developed tool.

## Data Availability

The datasets used and/or analyzed during the current study are available from the corresponding author on reasonable request.

## Abbreviations

AUC: Area Under Curves
CBCL: Child Behavior Checklist
EBP: Emotional and Behavioral Problems
METC: Medical Research Ethics Committee
PCH: Preventive Child Healthcare
PF: Parent Form
SDQ: Strengths and Difficulties Questionnaire
TDS: Total Difficulties Scale
TPS: Total Problem Scale
